# The need for improved Australian data on social determinants of health inequities

**DOI:** 10.5694/mja2.51495

**Published:** 2022-04-08

**Authors:** Joanne Flavel, Martin McKee, Toby Freeman, Connie Musolino, Helen van Eyk, Fisaha H Tesfay, Fran Baum

**Affiliations:** ^1^ Stretton Institute University of Adelaide Adelaide SA; ^2^ London School of Hygiene and Tropical Medicine London UK; ^3^ Institute for Health Transformation Deakin University Melbourne VIC

**Keywords:** Population health

Australia needs better data on health inequities to support building back fairer from the pandemic

The coronavirus disease 2019 (COVID‐19) pandemic has shone a light on longstanding inequities in societies.^1^ Yet, too often, these inequities are effectively invisible,^1^ and we can only know if we are tackling them if we can measure them. A lack of appropriate data is an important reason why research that has helped our understanding of health inequities is unevenly distributed internationally, with much concentrated in Europe and North America. Although Australia has some leading global centres for population health research, a lack of appropriate data creates a barrier to undertaking such research here. However, the available evidence indicates that socio‐economic health inequities have increased since the 1980s.^2^

A better understanding of what is happening is important for many reasons, not least the law of unintended consequences; policies designed to improve overall health can inadvertently widen health inequities.^3^ It is only by understanding the scale and nature of existing inequities and differential impacts of responses to them that we can assess the effect of policies and monitor progress.^4^ Improved data collection and analysis is the first essential step to building back fairer from the impacts of COVID‐19.

## Data gaps in Australia

The number of health data sources in Australia has grown since the late 1980s,^5^ but the 2012 Senate Standing Committee on Community Affairs inquiry on social determinants of health heard there were significant gaps in data on health inequities and social determinants of health that needed to be addressed through targeted research.^4^ A biennial Australian Institute of Health and Welfare (AIHW) publication on Australia’s health has repeatedly noted that there are data and analysis gaps in Australia that limit the monitoring of social determinants of health and their impacts on health equity.^6^ Crucially, this requires individual level data that capture the range of characteristics that lead to groups becoming disadvantaged, recognising in particular the concept of intersectionality where two or more characteristics may reinforce that disadvantage; for example, sexuality and ethnicity. In Australia, this includes not only Aboriginal and Torres Strait Islander peoples but also those from the many ethnic groups that contribute to an increasingly multicultural country, migrants and refugees, those with disabilities and from across the gender spectrum, and those in different geographical settings, from inner cities to remote rural communities.

The relatively poor capacity to collect and link health data in Australia has previously been noted by the Organisation for Economic Co‐operation and Development.^7^ The 2017 Productivity Commission 5‐year review noted: “while a huge amount of data are collected, there are substantial gaps, a lack of integration and sporadic use.”^7^ These gaps include, in particular, lack of data on health inequities and social determinants of health. Data collected on health focus on illness and disease rather than their underlying social determinants, limiting scope to ascertain causal effects of the distribution of social determinants on health equity. Many Australian health data collections do not include information on socio‐economic indicators or gather sufficient information on socio‐economic position to estimate the social gradient in health and the distribution of social determinants that contribute to the gradient. Although ad hoc surveys and population sub‐samples may provide insights at a national level, they miss the granularity needed to understand what is happening in individual states and territories, especially those that are smaller, and in different regions, especially those that are remote, within them.

In addition, where data are collected on health and socio‐economic variables, differing forms of measurement mean they are not comparable over time. This is true even for basic measures, such as income or education, which are not always measured consistently, while lack of data on other characteristics means they do not alone capture the multidimensional nature of socio‐economic status.^8^ Social determinants such as housing and food security are amenable to intervention and contribute to the gradient in health.^8^

Longitudinal data sources bridge some of these gaps (Box 1). Two key longitudinal datasets in Australia that collect health data are the Household, Income and Labour Dynamics in Australia Survey^9^ and the Australian Longitudinal Study on Women’s Health.^10^ The Household, Income and Labour Dynamics in Australia survey is now in its twenty‐first year of data collection and collects some health information and a wide range of data on social determinants of health. The Australian Longitudinal Study on Women’s Health is a long‐running project exploring factors contributing to health and wellbeing for four cohorts of Australian women. More recently, in 2013, Ten to Men: the Australian Longitudinal Study on Male Health^12^ began surveying a cohort of more than 15 000 boys and men, collecting information on health and social determinants of health. Current Australian longitudinal studies are primarily cohort studies which enable analysis of exposures and outcomes for the cohort included in the study. They are not designed for measurement of indicators of health equity and particularly the distribution of social determinants of health in the population, in states and territories, and in regional areas over time.

Box 1 **Longitudinal datasets in Australia that collect health information**

**Table:** 

Data source	Data collection commenced	Frequency of data collection	Target cohort	Strengths	Weaknesses
Household Income and Labour Dynamics in Australia Survey^9^	2001	Annual	A household‐based panel survey; population‐based sample of 17 000 individuals aged 15 years and over	Information is collected on some health indicators and the social determinants of health A breadth of information is collected	Although the wave 1 sample was selected to be representative of the population, the sample has become less representative with time Does not capture full distribution of social determinants (particularly at the top end) and limited data on health
Australian Longitudinal Study on Women’s Health^10^	1996	Varies by cohort; about every 3 years for most cohorts	Four cohorts of women born in specified years: 1921–1926; 1946–1951; 1973–1978; 1989–1995	Collects very detailed information on health and wellbeing of participants Long‐running survey and large sample	Limited collection of information on social determinants of health Information can only be used to identify distribution of health and social determinants within cohorts of interest
45 and Up Study^11^	2006	At least once every 5 years	250 000 NSW participants aged 45 years and over in 2006	Large sample Includes self‐report data and links to routinely collected health data Allows findings at state level (for NSW)	Limited collection of information on social determinants of health Can identify distribution of health and social determinants only for people aged 45 and over
Ten to Men: the Australian Longitudinal Study on Male Health^12^	2013	2 years between waves 1 and 2; 5 years between waves 2 and 3	Cohort of 15 000 males aged 10–55 years in 2013–14	Collects detailed information on health Also collects more information on social determinants compared with other studies	Does not collect information on all social determinants of health Information on distribution of health and social determinants only for males within cohort
Longitudinal Surveys of Australian Youth^13^	1995	Annual	Six cohorts aged 15–25 years; samples who were in school year 9 in 1995, 1998, 2003, 2006, 2009 and 2015	Collects information on many social determinants of health A breadth of information is collected	Limited information on health Information on distribution of health and social determinants only for young people aged 15–25 years
Longitudinal Study of Australian Children^14^	2003	Every 2 years	A representative sample of children from two cohorts aged 0–1 years in 2003‐04 and 4–5 years in 2003–04	Collects information on health and social determinants of health Later waves collect more information on young people’s health and collect information on social capital	Information on distribution of health and social determinants is for the subsection of the population in the two cohorts of interest
Longitudinal Study of Indigenous Children^15^	2008	Annual	Two cohorts of Aboriginal and/or Torres Strait Islander youth: aged 6–18 months in 2008 and 3.5–5 years in 2008	Collects information on a wide range of topics including physical and mental health, education, housing, culture and language, parental education, work and finances	Data collected is valuable for informing about the health of Aboriginal and Torres Strait islander children and is not intended to provide information on the distribution of health and social determinants for a wider population

Measurement of indicators of health equity and social determinants of health over time, nationally, for states and territories, and for regional and remote areas, is crucial to monitoring trends in the distribution of health and social determinants of health, and informing strategies that will reduce inequities.^16^ The Public Health Information Development Unit does admirable work in producing statistics on socio‐economic distribution of health and social determinants of health using the Index of Relative Socio‐Economic Disadvantage (IRSD), but the format of the data precludes assessment of causal pathways. The IRSD and other related indices of socio‐economic status of areas are also included in Australian Bureau of Statistics data sources, but as area level measures they do not enable analysis of individual level relationships.

A recent, positive development is the 2021 AIHW report on the impacts of the first and second waves of COVID‐19 on the population, health care system and social determinants of health.^17^ The report found that the lowest socio‐economic group had almost four times as many COVID‐19 deaths compared with the highest socio‐economic group.^17^ The AIHW acknowledged that negative social impacts of COVID‐19 have the potential to affect future population health.^17^ Further data are needed to monitor how these impacts on social determinants of health have affected health inequities.

## Data are necessary but are not sufficient to reduce health inequities

The Australian Health Performance Framework (AHPF) superseded the National Health Performance Framework and was endorsed by the Australian Health Ministers Advisory Council in 2017.^18^ However, as with its predecessor, it is not designed to support policy related performance against reducing inequities. The AIHW reports on the AHPF indicators in the biennial Australia’s Health report,^6^ but the Framework and related reporting are not linked to policies designed to reduce inequities. Adapting the AHPF to compel reporting on progress against indicators of health inequity would identify health equity and the collection of better data on inequities as a priority. It would also provide information on the consequences of government policies and identify underperformance.^1^

Previous Australian governments have used their powers to adopt policies that promote health equity,^19^ exemplified by the introduction of Medicare and paid parental leave. We obviously recognise that improved data that clearly demonstrate the structural causes of inequity alone will not lead to policies designed to reduce inequities. However, such data can be used by civil society and policy advocates to build political will for action to achieve health equity,^20^ and the data’s existence will be important in winning political will.

## Future directions

Although the data needed for health research^5^ and data linkage^21^ have improved, further improvements are possible and necessary in Australia.^6^ Data linkage for health research in Australia remains challenging, particularly for studies covering multiple jurisdictions.^21^ Evidence from elsewhere shows the potential that is unrealised, with Nordic countries among the international leaders.^22^ However, there are many other examples, such as those that exploit natural experiments in which policies are enacted in some areas, or with some groups, but not simultaneously in others, as in a Scottish study of urban renewal,^23^ a New Zealand study capturing the complex interplay between ethnicity, occupation and health,^24^ and a Swiss study that estimates the otherwise difficult‐to‐measure variable, wealth of pensioners.^25^

We recommend the establishment of a new, regularly collected, good quality data source on the distribution of health and social determinants of health that can be analysed nationally, in states and territories, and by remoteness (Box 2). This will enable monitoring of social determinants of health inequities in Australia in line with international best practice. Further data on social determinants of health and measures of the distribution of health could also be integrated into existing cohort studies, while streamlining and simplifying processes for data linkage would greatly assist researchers seeking to use data linkage to study health equity and social determinants of health.

Box 2 **Data to be collected**

▪ Representative samples for each state and territory, nationally and by remoteness
▪ Health data that capture distribution of health of individuals
▪ Data on social determinants of health: income, wealth, housing, education, employment, social inclusion/exclusion
▪ Data on disability, Indigenous status, and migrant status
▪ Data on ethnicity, culture and language, and social support to complement measures of socio‐economic status/position
▪ Neighbourhood characteristics: socio‐economic status of area of residence
▪ Data on gender, including non‐binary and transgender categories as well as female/male

The increasing health inequities evident in Australia and shown during the pandemic underline the importance of data on health inequities and social determinants of health. It is vital information for policy development and to elevate political and policy discussion of health inequities. Data alone will not force the hand of government to action, but as long as the causes remain invisible, action is much less likely to happen.

## Open access

Open access publishing facilitated by The University of Adelaide, as part of the Wiley ‐ The University of Adelaide agreement via the Council of Australian University Librarians.

## Competing interests

No relevant disclosures.

## Provenance

Not commissioned; externally peer reviewed.
